# Quinoa Abiotic Stress Responses: A Review

**DOI:** 10.3390/plants7040106

**Published:** 2018-11-29

**Authors:** Leonardo Hinojosa, Juan A. González, Felipe H. Barrios-Masias, Francisco Fuentes, Kevin M. Murphy

**Affiliations:** 1 Sustainable Seed Systems Lab, Department of Crop and Soil Sciences, College of Agricultural, Human, and Natural Resource Sciences, Washington State University, Pullman, WA 99164-6420, USA; l.hinojosasanchez@wsu.edu; 2 Facultad de Recursos Naturales, Escuela de Agronomía, Escuela Superior Politécnica de Chimborazo, Riobamba 060106, Ecuador; 3 Fundación Miguel Lillo, Instituto de Ecología, Miguel Lillo, San Miguel de Tucumán Post 4000, Argentina; jalules54@gmail.com; 4 Department of Agriculture, Veterinary and Rangeland Sciences, University of Nevada-Reno, Reno, NV 89557, USA; fbarrios@cabnr.unr.edu; 5 Facultad de Agronomía e Ingeniería Forestal, Pontificia Universidad Católica de Chile, Vicuña Mackenna, Macul, Santiago 4860, Chile; frfuentesc@uc.cl

**Keywords:** quinoa, abiotic stress, heat, drought, salinity, mechanism

## Abstract

Quinoa (*Chenopodium quinoa* Willd.) is a genetically diverse Andean crop that has earned special attention worldwide due to its nutritional and health benefits and its ability to adapt to contrasting environments, including nutrient-poor and saline soils and drought stressed marginal agroecosystems. Drought and salinity are the abiotic stresses most studied in quinoa; however, studies of other important stress factors, such as heat, cold, heavy metals, and UV-B light irradiance, are severely limited. In the last few decades, the incidence of abiotic stress has been accentuated by the increase in unpredictable weather patterns. Furthermore, stresses habitually occur as combinations of two or more. The goals of this review are to: (1) provide an in-depth description of the existing knowledge of quinoa’s tolerance to different abiotic stressors; (2) summarize quinoa’s physiological responses to these stressors; and (3) describe novel advances in molecular tools that can aid our understanding of the mechanisms underlying quinoa’s abiotic stress tolerance.

## 1. Introduction

Abiotic stress is the primary cause of crop losses, decreasing yields by more than 50% worldwide [1]. Many of these stressors naturally occur in combination. The main abiotic stresses—drought, waterlogging, high salinity, heavy metals, excess heat, frost, and ultraviolet-B light irradiance (UV-B)—have been extensively studied in plants [1,2,3,4,5,6,7,8]. The average annual global air temperature is expected to increase between 0.3 and 0.7 °C per decade, and by the end of this century, the highest predicted temperature increase approximates 4.8 °C due to climate change [9]. In this scenario, the predicted temperature extremes, or heat waves in summer, have received more attention due to their anticipated adverse impacts on human mortality, economies, and ecosystems [10,11].

**Figure 1:**
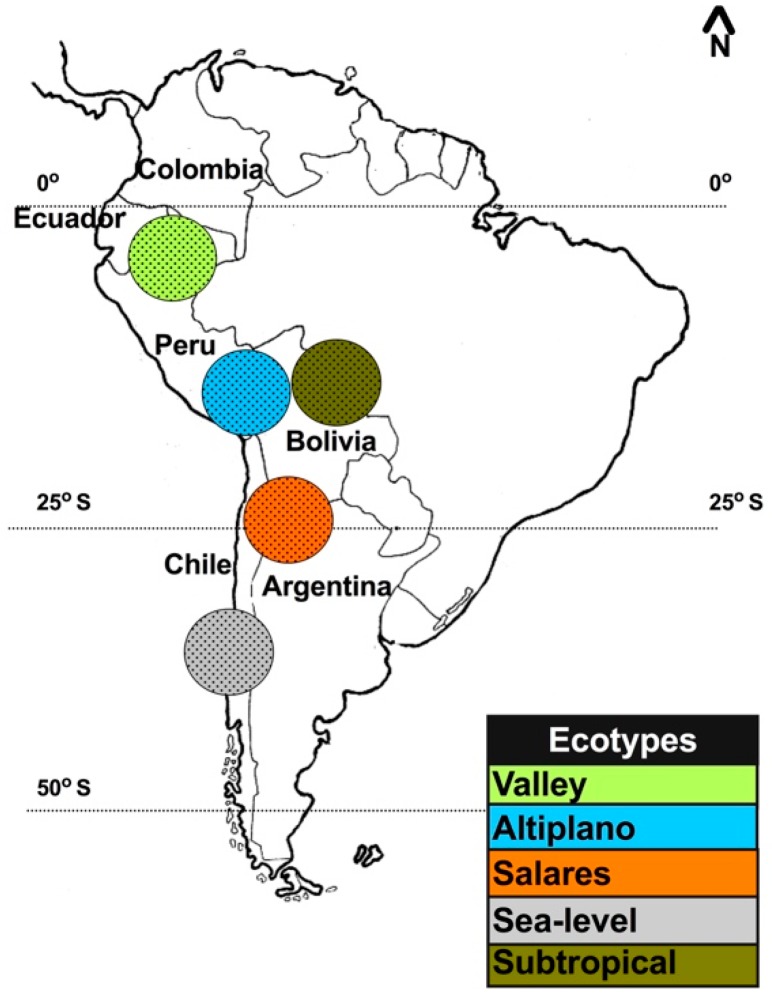
Geographic distribution of the five quinoa ecotypes.

Quinoa is an Andean crop, known as “kiuna” or “kinwa” in the Quechua language and “jupha” or “jiura” in the Aymara language [12]. Quinoa is widely cultivated, from sea level at the coast to 4000 m above sea level (m.a.s.l.). The plant’s natural geographical distribution ranges from southern Colombia (2 °N) to the coast of south-central Chile (43 °S), including a branch in northwest Argentina and some subtropical lowlands in Bolivia [13,14] (Figure 1). Originally, quinoa was domesticated in southern Peru and Bolivia close to Titicaca Lake and evidence of human cultivation dates back to between 8000 and 7500 years before present (B.P.) [15].

Quinoa is traditionally classified into five ecotypes, based on geographic adaptation, as follows: (1) valley = grown at 2000 to 3500 m.a.s.l. in Colombia, Ecuador, Peru, and Bolivia; (2) altiplano = grown at high altitudes of more than 3500 m.a.s.l. around Titicaca Lake on the border of Bolivia and Peru; (3) salares = grown in the salt flats of Bolivia and Chile and has a high tolerance to salinity; (4) sea-level = grown in the low-altitude areas of southern and central of Chile; and (5) subtropical or yungas = grown in the low-altitude, humid valleys of Bolivia and includes late-flowering genotypes (Figure 1) [12]. Quinoa germplasm is highly diverse. The natural variability in different traits, such as inflorescence type, seed color, seed size, life-cycle duration, salinity tolerance, saponin content, and nutritional value, allows quinoa to adapt to diverse environments [16,17,18,19,20,21,22,23]. To protect the genetic variability of quinoa in the Andean region, several gene banks have been created since the 1960s. A total of 16,422 accessions have been conserved in 59 gene banks across 30 countries, the majority of which are concentrated in Bolivia and Peru [23,24,25].

Quinoa is adapted to a wide range of marginal agricultural soils, including those with high salinity and those prone to drought. Recently, several papers have primarily addressed salt and drought tolerance in quinoa [14,26,27,28,29,30,31,32,33]. However, since the quinoa reference genome has been published [34], new transcriptome studies in salinity and drought in quinoa have been completed. Furthermore, information is limited about quinoa’s tolerance to other abiotic stress factors, such as frost, UV-B irradiance, and high air temperature. The purpose of this review is to give an overview of the current state of knowledge about quinoa tolerance to salt and drought, plus a variety of other abiotic stressors, namely high air temperature, UV-B radiation, frost, waterlogging, and heavy metal contamination. In particular, we discuss: (1) quinoa’s morphological, physiological, and molecular responses to these stressors; (2) management strategies to reduce the effects of these stressors; and (3) recent advancements in genetic and molecular resources that can help breeders improve quinoa’s tolerance to abiotic stress.

## 2. Drought

Agricultural drought is defined as the insufficient soil moisture that causes a reduction in plant production [35]. Quinoa is considered a drought-tolerant crop, capable of growing and producing seed grain in the semi-desert conditions of Chile, the arid mountain regions of northwest Argentina, and the Altiplano area of Peru and Bolivia. These environments are characterized as extremely arid, with less than 200 mm of annual rainfall [19,36,37,38,39,40,41]. Quinoa can also adapt and produce seed in semi-arid and arid environments outside of the Andean region, such as Asia, North Africa, the Near East, and the Mediterranean [28,42,43,44,45]. 

Although quinoa is inherently drought tolerant, different climatic models predict an increase in drought frequency, especially in the altiplano region of the Andes, where quinoa is grown traditionally by small farmers [46]. Thus, understanding the drought response mechanisms in quinoa is critical for developing varieties with improved drought tolerance.

### 2.1. Drought Response Mechanisms in Quinoa

Plants develop different response mechanisms to endure a lack of water. These mechanisms can be divided into three groups: (1) morphological strategies, such as avoidance, for instance, deeper roots, and phenotypic flexibility related to ontogenic processes that can contribute to the scape and avoidance strategy; (2) physiological strategies, such as antioxidant defense, cell membrane stabilization, plant growth regulation, stomatal conductance, and osmotic adjustment; and (3) molecular strategies, such as activating stress proteins (osmoprotectants) and aquaporins [47]. 

Quinoa’s flowering and milk grain stages have been established as the most drought-sensitive [38]. Several studies have been conducted to understand the quinoa plant’s mechanisms under drought stress [39,48,49,50,51,52,53,54]. In a pot experiment under drought conditions, Jacobsen et al. (2009) reported an increased concentration of abscisic acid (ABA) in the roots of quinoa altiplano variety ‘INIA-Illpa’, which induced a decreased turgor of stomata guard cells [52]. The same mechanism was observed in the leaves of sea-level variety ‘Titicaca’ when plants were grown under water deficient and control conditions [51,54]. Furthermore, during drought stress of ‘Titicaca’, the concentration of xylem ABA increased faster in the shoots than the roots [55]. Similar results were observed again in ‘Titicaca’ and altiplano variety ‘Achachino’; xylem sap ABA concentration increased two days after drought treatment and decreased to the control levels after re-watering. Under the drought conditions, ‘Titicaca’ had higher ABA concentrations than ‘Achachino’ [56].

Other drought response mechanisms in quinoa are the synthesis of reactive oxygen species (ROS) scavengers; accumulation of osmolytes as an antioxidant defense, particularly ornithine and raffinose pathways; and the accumulation of soluble sugars and proline, which adjust cellular osmotic potential [49,57,58,59]. Quinoa also develops response mechanisms to reduce water loss through rapid stomatal closure, cellular water deficit regulation, and root-to-shoot ratios that trigger a high water-use efficiency [50,51,52,54,55,56,60,61]. However, Jensen et al. (2000) found that quinoa altiplano variety ‘Kankolla’ was insensitive to drought relative to stomatal response at early growth stages [53]. In response to this finding, they proposed that high net photosynthetic rates and a specific leaf area in early growth stages support water uptake by larger root systems that helps the plant avoid drought later on. Other drought response mechanisms could involve a delay in development when drought was imposed at the pre-anthesis stage under Bolivian Altiplano conditions [62].

One of the major effects of drought on plants is a reduction in the photosynthetic rate, which is primarily due to stomatal closure [63]. Leaf gas exchange and carbon isotope discrimination (Δ) are common approaches used to study plants under drought conditions [64]. González et al. (2011) evaluated leaf gas exchange and Δ^13^C in 10 quinoa genotypes grown in the arid mountain region of northwest Argentina, which receives 160 mm of rainfall during the growing season [39]. Results showed that quinoa genotypes with higher stomatal conductance were capable of maintaining higher photosynthetic rates. Additionally, the researchers observed high variability in the grain yield among genotypes and found a positive correlation between Δ^13^C and yield. Similar results were reported by [60], where drought-induced quinoa experienced pronounced stomatal and mesophyll limitations to CO_2_ transport. However, in quinoa greenhouse experiments, indicators of leaf photosynthetic capacity, such as the maximum quantum yield of PSII (F_v_/F_m_) and quenching analysis (qP and qN), were insensitive to water stress [50,60,65]. On the other hand, F_v_/F_m_ decreased in response to drought effects in the variety ‘Titicaca’ in a greenhouse experiment [54].

During two successive quinoa growing seasons in Morocco, field experiments used OJIP analysis, defined by the O, J, I, and P steps that correspond to the redox states of the photosystem (PS II and PS I); this analysis explores changes in the photosystem II (PSII) photochemical performance [66]. Results showed that drought stress in a sea-level grown ‘Puno’ variety induced a decrease in F_v_/F_m_ and in the quantum yield of electron transport (φE0), proposing OJIP parameters as a viable drought stress evaluation tool. Conversely, another analysis of chlorophyll a fluorescence OJIP transient in the sea-level variety ‘Red Head’, grown in semi-controlled conditions in Italy, revealed no difference in 16 chlorophyll fluorescence parameters between control and drought treatments [60]. These two contradictory studies likely demonstrate genotypic variability in the quinoa response to drought. Consequently, additional studies that include a greater number of genotypes and simultaneous measurements of gas exchange are required to establish chlorophyll a fluorescence OJIP transient’s effectiveness as a drought evaluation tool for quinoa.

Finally, root system architecture and its relationship to soil moisture conditions has been studied in quinoa and quinoa relatives. Quinoa roots exhibit faster elongation and abundant and longer external branching of the roots that improve their foraging capacity compared to quinoa relatives *C. hiricinum* and *C. pallidicaule* [67,68]. Recently, a root system architecture and dynamics study was conducted in drought conditions, comparing *C. hiricinum* and *C. pallidicaule*, a rainy-habitat and a dry-habitat quinoa genotype, respectively. Results showed that the quinoa genotypes exhibited accelerated taproot growth in dry soil conditions compared to the other two species. Furthermore, the quinoa genotype from the dry habitat showed longer, coarser, and more numerous root segments than the wet-habitat genotype [48]. These findings led the authors to suggest quinoa as a promising plant model to investigate biophysical and ecophysiological traits of plant rooting in deep soil layers.

### 2.2. Field Studies Under Drought Conditions 

In Italy, Pulvento et al. (2012) found that yields for ‘Titicaca’ ranged between 2.30–2.70 t ha^−1^ whether grown under high irrigation (300–360 mm) or deficit irrigation (200–220 mm) during the growing season [44]. Thus, the study concluded no significant yield reductions due to a lack of water. Similar results in the yield with the same variety were observed in Demark when plants were grown under irrigated and non-irrigated conditions in sand, sandy loam, and sandy clay loam soils [69]. However, in Egypt, five quinoa genotypes were evaluated under three different growing season water regimes, consisting of high (820 mm), moderate (500 mm), and low (236 mm) irrigation treatments (rainfall + irrigation). Results showed high variability in morphological traits and yield among genotypes across the different water regimes. For instance, sea-level variety ‘QL-3’ exhibited the biggest reduction in yield (56%) under severe water stress; valley variety ‘CICA-17’ showed the smallest reduction (12%) [70].

Quinoa yield reductions under dryland conditions were reported for sea-level varieties ‘Cherry Vanilla’ and ‘Oro de Valle’ when grown in an organic field in Pullman, WA—an area characterized by dry, warm summers [71]. This study was carried out under three intercrop treatments (clover/medic mix, fescue grass/clover mix, and a no intercrop control) and three irrigation regimens (dryland, 64 mm, and 128 mm of water). Results showed that neither of the two intercrop treatments affected the quinoa yield under irrigated or non-irrigated conditions. However, irrigation can relieve the effect of high temperature; for instance, the mean yield under the dryland regime increased from 0.2 t ha^−1^ to 1.2 t ha^−1^ with an extra 128 mm of water during the growing season [71].

### 2.3. Irrigation Strategies to Mitigate Drought Stress

The crop coefficient (K_c_) is the ratio of evapotranspiration of a crop to a reference crop, such as a perennial grass. K_c_ helps predict crop irrigation needs in different phenological development stages using meteorological data from a weather station [72]. To estimate irrigation requirements for the quinoa altiplano variety ‘Chucapaca’ grown in the Bolivian Altiplano, Garcia et al. (2003) calculated K_c_ as 0.52, 1.00, and 0.70 for the initial, mid-season, and late-season phenological stages, respectively [73]. In contrast, K_c_ values for the variety ‘Titicaca’ grown in Demark were higher than values reported for the Bolivian Altiplano, equaling 1.05, 1.22, and 1.00 for initial, mid-season, and late-season stages, respectively [69]. Full irrigation to increase the quinoa yield in water-scarce regions is not an option; however, partial root zone drying and deficit irrigation are practices that reduce the amount of water used during the growing season without detriments to yield and might be useful alternatives [38,74]. Nevertheless, in regions with poor quality water (saline groundwater table), deficit irrigation must be managed with caution to avoid high salt accumulation in the root zone [75].

### 2.4. Other Drought Stress Mitigation Strategies

In addition to irrigation, several other approaches for relieving drought stress in quinoa have been studied. For example, greenhouse experiments with ‘Titicaca’ showed that applications of nitrogen (N) supplied as ammonium nitrate (NH_4_NO_3_) at a rate of 0.6 g N plot^−1^ could improve plant performance under water stress. Observed drought tolerance mechanisms included faster stomatal closure, lower leaf water potential, and higher leaf ABA concentrations [76]. Other studies of drought stressed quinoa have found that adding compost and acidified biochar to soils under drought conditions can improve quinoa plant growth, yield, physiological, and antioxidant activity, and chemical and biochemical attributes of quinoa seeds [77,78,79,80]. For example, under field conditions in Morocco, organic amendments can relive the drought effect in quinoa; yields increased from 1.7 to 2.0 t ha^−1^ using 10 t ha^−1^ of compost under non-irrigated conditions [78,79]. Similar increases in yield were observed for two quinoa genotypes grown in the semi-arid conditions of Chile when vermicompost was used to enhance soil organic matter [40]. The yield increased from 5.8 g plot^−1^ to 9.4 g plot^−1^ by adding acidified biochar in drought conditions [80,81].

Moreover, a study with quinoa genotype ‘V_9_’, subjected to varying irrigation regimes, demonstrated that foliar applications of 150 mg L^−1^ synthetic ascorbic acid and 25% concentration of orange juice (natural ascorbic acid) diluted in distilled water mitigated the harmful effects of drought stress in quinoa [77]. Plant growth, total carotenoids, free amino acids, and several antioxidant enzymes increased due to synthetic ascorbic acid and orange juice in drought conditions [77]. Exogenous ascorbic acid protects lipids and proteins from the plants against drought-induced oxidative adversaries [82]. Proline was used as another foliar treatment under field conditions in Egypt [83]. Results showed that foliar applications of 12.5 mM and 25.0 mM of proline improved growth parameters, relative water content, yield components, and nutritional values. Applications of 25.0 mM of proline increased the yield from 6.23 g plant^−1^ to 8.56 g plant^−1^ in drought conditions. Additionally, a pot experiment under greenhouse conditions using the quinoa sea-level variety ‘Pichaman’ showed that applying 80 mM of exogenous H_2_O_2_ as a seed primer and 15 mM as a foliar spray improved the quinoa performance under drought conditions. For instance, plants exhibited higher photosynthetic rates, stomatal conductance, chlorophyll content indices, proline levels, sugar contents, and ABA regulation [84]. Exogenous H_2_O_2_ acts as an oxidative modifier and mobilizer of stored proteins [85].

Unique fungal-root associations in quinoa may aid in the plant’s ability to tolerate drought conditions. Several studies have characterized the endophytic fungi associated with quinoa roots and bacterial endophytes in quinoa seeds [86,87,88,89]. Quinoa roots were collected in natural conditions close to the Salt Lake of the Atacama Desert in Chile. Molecular analysis showed that quinoa roots shelter a diverse group of endophytic fungi. *Penicillium, Phoma*, and *Fusarium* genera dominated the fugal community [90]. The fungus *Penicillium minioluteum,* isolated from the characterization described above, was used to study the effects of root endophytic fungi on drought stress in a quinoa variety from the Atacama Desert. Results demonstrated a 40% improvement in root biomass relative to the treatment with no inoculum. However, the study found no improvement in photosynthesis, stomatal conductance, or photochemical efficiency by the presence of the endophytic fungi. Thus, the interaction between *P. minioluteum* and quinoa exhibited a positive response in root biomass, but only under drought conditions [86]. Another study was conducted using the root endophyte *Piriformospora indica* and the quinoa valley variety ‘Hualhuas’ under greenhouse conditions. Results showed the successful colonization of *P. indica* in quinoa. This association could mitigate some drought effects by improving the plant water and nutrient status, resulting in the capacity to increase total biomass, stomatal conductance, leaf water potential, and net photosynthesis [87].

### 2.5. Seed Quality Under Water Limitations

Environmental and climatic factors influence the nutritional quality of quinoa seeds. Variations in amino acids, protein content, mineral composition, and phytate were observed in 10 quinoa varieties between two semi-arid locations, northwest Argentina and the Bolivian Altiplano [91]. The interaction between genotype and environment (G × E) was responsible for mineral composition, amino acids, and protein variations among quinoa varieties [91,92]. Similar results were found with three quinoa varieties planted in Chile, Argentina, and Spain. For example, seed quality was primarily dependent upon G × E, with the exception of saponin and fiber content, which were more stable across locations [93]. 

In another study with two sea-level varieties, ‘Cherry Vanilla’ and ‘Oro de Valle’, seed protein content increased when quinoa was grown with irrigation and a clover-medic mixture intercrop system, compared to the same intercrop system without irrigation. Furthermore, the irrigated plants exhibited increased seed concentrations of P, Mg, and Fe, but decreased concentrations of Ca, Cu, and Zn, compared to the non-irrigated treatment [71]. On the other hand, Pulvento et al., (2012) found no differences in any aspect of seed quality in ‘Titicaca’ among three different irrigation treatments. However, seed fiber and saponin content increased when the quinoa plants were well-irrigated, compared to plants without irrigation [44].

In the south-central zone of Chile, seed quality was evaluated in quinoa sea-level varieties ‘Regalona’, ‘AG2010’, and ‘B080’, grown under four regimes of water availability in both greenhouse and field experiments. Results showed an increase in the seed antioxidant capacity of all three varieties and a minimal reduction in seed yield in ‘AG2010’ under 20% soil water availability relative to 95% soil water availability [94]. Moreover, 20% soil water availability in ‘AG2010’ increased globulin content, and the effect of washing quinoa seeds with water changed the concentration and electrophoretic pattern of albumins and globulins [95].

### 2.6. Gene Expression Under Water Limitation

Raney et al. (2014) performed the first RNA sequencing (RNA-seq) transcriptome analysis on quinoa under drought conditions with two varieties: valley variety ‘Ingapirca’ and salares variety ‘Ollague’. ‘Ollague’ demonstrated a greater drought tolerance compared to ‘Ingapirca’, based on several physiological parameters, including stomatal conductance, photosynthetic rate, and stem water potential [96]. RNA-seq using root tissue from both varieties under a control and different water stress treatments, identified 462 differentially expressed contigs and 27 putative genes with regulatory functions based in the interaction terms. Several of these 27 genes have unknown protein functions. However, other genes, such as AUR62041909 and AUR62015321, have known functions. Specifically, gene AUR62041909 functions as an intermediate in the biosynthesis of flavonoids in plants. Gene AUR62015321 belongs to the dirigent family of proteins, which are induced during the disease response in plants and are involved in lignification [97].

Heat-shock proteins (HSPs) have been studied since the 1960s as one of the stress-inducible proteins under several types of stress, but mostly under lethal temperatures [98]. In recent decades, HSPs have gained more attention as molecular chaperones, preventing the accumulation of other proteins and playing an important role in protein folding [99]. HSP superfamilies are grouped based on molecular weight, for example, HSP100, HSP90, HSP70, HSP60, and small heat-shock proteins (sHSPs) [98]. The HSP70s are not only up-regulated during heat stress conditions in plants, but also play an important role in response to other stresses, such as drought [100]. Recently, Liu et al., (2018) identified and characterized 16 quinoa HSP70 members (*Cqhsp70s)* in the newly sequenced quinoa genome [34], based on HSP70s in *Arabidopsis* [101]. Their study analyzed the expressions of 13 *Cqhsp70s* genes under drought conditions induced by polyethylene glycol 6000 (PEG6000). Results showed a significant variation in the gene response to drought stress. For instance, the expression of six out the 13 *Cqhsp70s* genes was down-regulated at the beginning of drought stress and during the recovery time. In another example, the expression of the gene *AUR62024018* remains high throughout the duration of the drought treatment. Moreover, one-half of the genes evaluated exhibited a “drop-climb-drop” expression pattern, which was similar to the homolog genes in *Arabidopsis*. 

In other work, Morales et al., (2017) studied the transcriptional responses of quinoa under drought stress [102]. First, they found that the salares variety ‘R49’ presented the highest drought tolerance compared to ‘PRJ’ and BO78 sea-level varieties; ‘R49’ displayed the best performance on physiological parameters, such as relative water content, electrolyte leakage, and (F_v_/F_m_). Second, RNA-seq was carried out on ‘R49’ under control and drought conditions. Fifty-four million reads were obtained for the control and 51 million for drought conditions. All reads were assembled into 150,952 contigs; 19% of genes (306 contigs) were not represented in published databases of homologous genes. Fifteen target genes were selected to analyze gene expression. Some of these genes were selected based on other plant models in which these genes have been induced under drought stress, focusing on ABA biosynthesis and ABA transport pathway functions. Other target genes were selected based on those that exhibited changes of representation reads by RNA-seq in quinoa. Results showed that just two genes associated with ABA biosynthesis, CqNCED3a and CqNCDE3b, which are localized in plastids, were up-regulated in response to drought in quinoa. Moreover, all genes that exhibited changes from representation reads, *CqHSP20* (putative chaperones hsp20- protein superfamily), *CqCAP160* (cold acclimation protein 160), *CqLEA* (late embryogenesis abundant protein family protein), *CqAP2/ERF* (integrase-type DNA-binding protein superfamily), *CqPP2C* (protein phosphatase protein family 2c), *CqHSP83* (chaperone protein, protein family HTPG), and *CqP5CS* (delta 1-pyrroline-5-carboxylate synthase 2), were up-regulated. In particular, genes CqHSP20 and CqLEA were altered over 140-fold expression. Both HSP studies described above concur that HSPs play an important role in the adaptation of quinoa under drought stress. Thus, quinoa could be an excellent model species to study HSPs under multiple stresses, such as drought, heat, and salinity. 

## 3. Salinity

Salinity refers to the presence of the major dissolved inorganic solutes, mainly Na^+^, Mg^2+^, Ca^2+^, K^+^, Cl^−^, SO_4_^2−^, HCO_3_^−^, and CO_3_^2−^. Soil salinity refers to the soluble plus readily dissolvable salts in the soil or in the aqueous extract and is quantified as the total concentration of the salts or by measuring the electrical conductance (EC) of a saturation soil extract. Soils are considered saline when EC is more than 4 dS m^−1^ at 25 °C [103]. The response of quinoa to salinity has been studied intensely during the last 20 years, prior to May 2018. Over 120 studies on the relationship between salinity and quinoa were published between 1998 and 2018; of these, approximately 60% of the studies were published within the last five years. Three extensive reviews about quinoa as a model for understanding salt tolerance have been published [26,33,104]. In the following, we briefly summarize the response mechanisms associated with quinoa’s salt tolerance and then detail new advances in gene transcription for understanding quinoa’s response to high salinity.

High salinity is considered one of the major abiotic stresses that limit crop yields because it causes reduced photosynthesis, respiration, and protein synthesis. Photosynthesis reduction, membrane denaturalization, nutrient imbalance, stomatal closure, and a dramatic increase in ROS production are the main physiological changes in plants under salinity stress [105,106,107]. High accumulations of ROS cause serious plant toxicity, including oxidative damage in proteins, lipids, and DNA; whereas, low concentrations of ROS act as signaling molecules [108,109,110,111].

### 3.1. Tolerance and Effects of High Salinity in Quinoa

Quinoa has been identified as a facultative halophyte crop, with greater salt tolerance than barley, wheat, and corn [112,113,114], as well as vegetable crops, such as spinach, carrots, onion, and even asparagus [115]. A high variability in salinity tolerance among quinoa genotypes has been reported [20,116,117,118,119]. Traditionally, only genotypes from the Bolivian Salares were thought to have a high tolerance to salinity [12]. However, salinity tolerance in quinoa does not correlate with geographic distribution; varieties from coastal regions of Chile and highland areas outside the Salares ecoregion have similar or even higher salt tolerance levels [113,118,120,121,122]. Additionally, a wild relative of quinoa (*Chenopodium hircinum*) was found to have a much higher salinity tolerance level than quinoa cultivars [118]. 

In general, quinoa can tolerate moderate to high levels of salinity, ranging from a salt concentration of 150 mM NaCl (electrical conductivity ~15 dS m^−1^) to as much as 750 mM NaCl (electrical conductivity ~75 dS m^−1^) [120], which is greater than the salinity of seawater (>45 dS m^−1^) [26]. In contrast, yields for glycophyte crops, such as wheat, rice, corn, and peas, start declining when the soil solution exceeds 40 mM NaCl (electrical conductivity ~4 dS m^−1^) [112,114,123].

The optimal salinity conditions for quinoa growth are between 100 to 200 mM NaCl [123,124,125]. Plant seedling and germination stages are the most sensitive to salinity, even for halophytes [126,127]. Salt concentrations between 100 to 250 mM NaCl do not affect germination rates in most quinoa genotypes [95,117,125,128,129,130,131,132]. However, concentrations between 150 to 250 mM NaCl delay the onset of germination [117,120,130]. Changes in invertase activity and soluble sugar metabolism have also been detected during the quinoa germination process under saline stress [130,133]. At the seedling stage, the sugar concentration can increase or decrease, depending on the genotype, in both cotyledons and roots when grown under 200 to 400 mM NaCl.

The osmotic stress produced by a high salt concentration increases ABA production in roots and subsequent transport to the leaves as a signal to regulate stomatal conductance. The closure of stomata reduces water loss but also CO_2_ uptake, thereby inhibiting photosynthesis [134]. Different experiments in semi-controlled conditions under salinity treatments have been conducted in quinoa to evaluate photosynthesis. In two quinoa varieties, salares variety ‘Utusaya’ and ‘Titicaca’, the CO_2_ assimilation was reduced to 25% and 67%, respectively, when plants were grown at 400 mM NaCl [135]. In the altiplano variety ‘Achachino’, an increase of salinity from fresh water to 250 mM NaCl reduced the net assimilation rate of photosynthesis from 30 μmol CO_2_ m^−2^ s^−1^ to 10 CO_2_ μmol m^−2^ s^−1^ in a Photosynthetic Active Radiation (PAR) level of 1500 μmol m^−2^ s^−1^ [136]. Another experiment with the valley variety ‘Hualhuas’ showed a 70% reduction in the net photosynthetic rate when plants were grown under a salinity level of 500 mM NaCl [137]. Saline groundwater treatments with 100, 200, 300, and 400 mM NaCl were used in an experiment ‘Titicaca’. Results showed that increasing the water salinity from 100 to 400 mM NaCl reduced the net assimilation rate of photosynthesis by 48% and seed yield by 72% [138]. On the other hand, the elevated atmospheric CO_2_ (540 ppm) mitigated the effect of high salinity by tempering the stomatal limitation effect on photosynthesis, and consequently, reducing the hazard of oxidative stress [139].

The variety ‘Titicaca’, grown in field conditions under 22 dS m^−1^ and limited water in a Mediterranean environment, exhibited no yield reduction [44,51]. In another experiment with the same variety, the seed yield was reduced by 32% when plants were grown under 40 dS m^−1^ compared to the control (0 dS m^−1^) [69]. However, under the same Mediterranean conditions, the sea-level variety ‘Red head’ grown under 30 dS m^−1^ exhibited a high susceptibility to salinity; various physiological parameters such as photosynthesis were affected [60].

Recently, new approaches such as halotolerant rhizobacteria and seed priming have been studied as alternatives to improve quinoa’s physiological response to salinity stress [140,141]. For example, plant growth-promoting rhizobacteria have been used to alleviate the damage caused by salt stress because of their ability to fix nitrogen, produce siderophores, dissolve mineral insoluble phosphate, and produce phytohormones [142]. Seed priming partially hydrates seeds to the point of initiating the germination process. Treated seeds are then usually re-dried before planting. Different priming techniques are available, depending on which seed-embedding substance is used [143].

Yang et al. (2016) studied the relationship between plant growth-promoting halotolerant bacteria (*Enterobacter* sp. and *Bacillus* sp.) and quinoa under saline conditions [140]. Results showed that both strains mitigated the negative effects of salinity, reducing Na^+^ uptake and improving water relations when the plants were grown at 300 mM NaCl. The same research group demonstrated that using saponin as a seed primer bio-stimulated quinoa germination under 400 mM NaCl [141]. In addition, seed priming ‘Titicaca’ with water (hydropriming) and with polyethylene glycol (osmopriming) showed that both hydropriming and osmopriming improved germination in salinity conditions [144].

Paclobutrazol, a gibberellic acid biosynthesis inhibitor, has been used to increase the yield and reduce plant height in quinoa [145]. More recently, Waqas et al. (2017) used this approach to mitigate salt stress in quinoa. They applied paclobutrazol (20 mg/L) on the leaves of sea-level variety ‘Pichaman’ under high salinity conditions (400 mM NaCl). Results showed improved chlorophyll and carotenoid content, enriched stomatal density on both leaf surfaces, and increased accumulation of osmoprotectants and antioxidants in leaf and root tissues [146]. All approaches described above could be excellent alternative tools to improve the quinoa yield in high salinity conditions.

### 3.2. Epidermal Bladder Cells and Stomatal Density

Morphological traits such as stomatal density and epidermal bladder cells (EBCs) have been studied in quinoa under salinity stress [119,120,136,147,148,149,150,151]. EBCs are modified epidermal hairs and classified as trichomes, along with glandular hairs, thorns, and surface glands. EBCs are shaped like gigantic balloons, with a diameter that is around 10 times bigger than epidermal cells, and can sequester 1000-fold more Na^+^ compared with regular leaf cell vacuoles. EBCs accumulate water and different metabolites, such as betaine, malate, flavonoids, cysteine, inositol, pinitol, and calcium oxalate crystals. The main function of calcium oxalate is to regulate calcium levels and protect the plant against herbivores [152]. EBCs likely play a role in plant tolerance to ultraviolet (UV) light because they accumulate betacyanins and flavonoids, which are associated with UV protection and water homeostasis functions [153,154]. EBCs in *Chenopodium* species have a defensive function against herbivore insects and serve as structural and chemical components of defense [155]. EBCs play important roles in halophyte plants, such quinoa and Atriplex species. Their primary roles are the sequestration of sodium, improved K^+^ retention, and the storage of metabolites, which help to modulate plant ionic relations, mainly gamma-aminobutyric acid [147].

**Figure 2:**
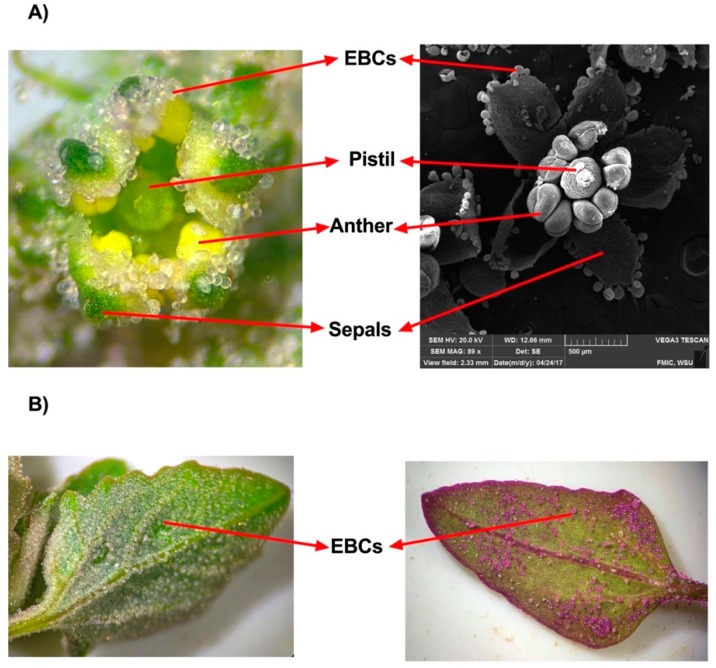
Epidermal bladder cells (EBCs). (**A**) EBCs localized in the quinoa flower. Left, micrograph of quinoa flower. Right, scanning electron micrograph of quinoa flower. (**B**) Micrograph of quinoa leaves in two different varieties of EBCs.

In quinoa, EBCs are localized in leaves, stems, and inflorescences (Figure 2A,B). EBC density does not increase in response to high salinity [120,136]. However, the number of EBCs is greater in young leaves than in old leaves [135,156]. High sequestration of Na^+^ in the EBCs from young leaves of quinoa under saline conditions (400 mM NaCl) has been reported [156]. Recently, with the assembly of a draft quinoa genome for the salares variety ‘Real’, transcriptome sequencing on bladder cells under both salt-treatment (100 mM NaCl) and non-treatment conditions has been conducted [148].

Stomatal area and density have been studied in quinoa under a variety of salinity conditions [120,136,151]. A salinity concentration of 400 mM NaCl was shown to reduce the number of stomata per leaf area in young, intermediate, and old leaves in ‘Titicaca’ [151]. Similar results were observed in the Chilean sea-level variety ‘BO78’; the highest reduction in stomatal density (54%) was under 750 mM NaCl, compared with the untreated control [120]. Another study with 14 quinoa varieties by Shabala et al., (2013) demonstrated that stomatal densities in all varieties decreased when plants were grown in 400 mM NaCl [119]. Furthermore, the study found a strong positive correlation between stomatal density and plant salinity tolerance. Opposite results were observed in ‘Achachino’, where stomatal density increased ~18% when the plants were grown at 250 mM NaCl; nonetheless, stomatal size was reduced by the salinity effect [136]. Stomatal density and size could be a key mechanism for optimizing water-use efficiency under saline conditions.

### 3.3. Mechanisms of Salt Tolerance

Several studies to identify mechanisms of salt tolerance in quinoa have been conducted over the last 15 years. Quinoa has diverse mechanisms to withstand high levels of salt, including the efficient control of Na^+^ sequestration in leaf vacuoles, xylem Na^+^ loading, higher ROS tolerance, better K^+^ retention, the upkeep of low cytosolic Na^+^ levels, the reduction of slow and fast tonoplast channel activity, and a high rate of H^+^ pumping in the mesophyll cell [26,33,156,157].

The accumulation of compatible solutes, such as proline and total phenolics, is associated with salt tolerance in quinoa [51,59,125,131,132,158,159]. However, Ismail et al., (2016) showed that proline may not play a major role in either osmotic adjustment or in the tissue tolerance mechanism [160]. But, the non-enzymatic antioxidant rutin improves quinoa salinity tolerance by scavenging hydroxyl radicals. Choline (Cho^+^) is a metabolic precursor for glycine betaine and plays an important role in the osmotic adjustment to salinity stress in quinoa [161]. Polyamines were studied in four Chilean varieties under control (0 mM NaCl) and 300 mM NaCl conditions. The total amount of polyamines was reduced under the salinity conditions; however, the ratio of (sperdimidine+spermine)/putrescine increased up to 10-fold [132]. In addition, the activity of antioxidant enzymes changes in response to salinity in quinoa. For example, Panuccio et al. (2014) observed that, in quinoa seedlings of ‘Titicaca’, the regulation of antioxidant enzymes depended on the type and concentration of salt [129]. NaCl resulted in higher activity levels of superoxide dismutase (SOD), peroxidase (POX), ascorbate peroxidase (APX), and catalase (CAT), compared to the other salts evaluated (KCl, CaCl_2_, MgCl_2_). The exception was seawater from the Tyrrhenian Sea, which produced a major increase in POX, APX, and CAT activity [129].

High concentrations of NaCl can generate K^+^ and H^+^ fluxes in quinoa roots to the apoplast; thus, the activation of plasma membrane H^+^-ATPase is needed to avoid further K^+^ leakage from the cytosol [120,125]. H^+^-ATPase is one of the active transporters, along with channels and co-transporters, that maintain intracellular K^+^ and Na^+^ homeostasis [162]. An analysis of cytosolic Na^+^ showed that Na^+^ is removed quickly from the cytosol. The high K^+^ concentration in both roots and shoots permitted higher pump activity when plants were grown under moderate salinity conditions [123]. Likewise, Cho^+^ blocks the tonoplast slow vacuolar channels in quinoa leaf and root tissue, triggering efficient Na^+^ sequestration [161].

Recently, the activity of mitogen-activated protein kinase (MAPK) under saline conditions (400 mM NaCl) was studied in quinoa seeds and seedlings. MAPK activities were highest in seeds and decreased during germination. Changes in MAPK activities occurred soon after imbibition, either with water or salt. Furthermore, under the salinity conditions, the decrease in MAPK activity occurred sooner than in non-stressed conditions [89].

### 3.4. Seed Composition Under Salinity Conditions

A few studies have reported changes in seed quality under saline conditions. The nutritional quality of 10 varieties of quinoa (nine from the Bolivian Altiplano and one from the Northwest Andean region of Argentina) was evaluated in two locations: Encalilla-Argentina (electric conductivity 2 dS m^−1^) and Patacamaya-Bolivia (electric conductivity 7 dS m^−1^). Results demonstrated that essential amino acids were affected more by salinity than yield and protein level. Seven of the 10 varieties studied exhibited increased essential amino acids when grown in the higher salinity location [91].

Quinoa varieties ‘Titicaca’ and ‘Q52’ were evaluated under field conditions in Italy using different irrigation regimes under saline conditions (22 dS m^−1^). Results showed that seed fiber and saponin content decrease in response to the highest level of saline water; however, protein content was unaltered [44,163]. Similar results in seed fiber were found in the variety ‘Hualhuas’ under field conditions of 17.9 dS m^−1^ in the northwestern part of Sinai-Egypt [124]. On the other hand, seed protein content in eight different varieties increased under a saline-sodic soil (6.5 dS m^−1^) in Larissa-Greece [164]. Similarly, four sea-level quinoa varieties, consisting of ‘CO407D (PI 596293)’, ‘UDEC-1 (PI 634923)’, ‘Baer (PI 634918)’, and ‘QQ065 (PI 614880)’, exhibited increased seed protein content when grown under 32 dS m^−1^ Na_2_SO_4_ conditions. In contrast, the study found no change under the same concentration of NaCl [165].

Prado et al. (2014) found variation in the concentrations and tissue distributions of 18 mineral elements in the seeds of seven different quinoa cultivars from Patacamaya, Bolivia (3960 m above sea level) and from Encalilla, Argentina (1980 m above sea level) [92]. The data clearly showed inter- and intra-varietal differences in seed mineral concentrations between the two sites, strongly suggesting that G × E interactions were responsible for mineral variation among the quinoa cultivars. In another study, the mineral content of calcium (Ca), magnesium (Mg), zinc (Zn), and manganese (Mn) in quinoa seeds decreased in response to saline-sodic soil in Larissa-Greece [164]. Similar results were found for the valley variety ‘Hualhuas’ under field conditions, with 17 dS m^−1^ in the northwestern part of Sinai-Egypt [124]. Using X-ray microanalysis, the study found high Na^+^ accumulation in the pericarp and embryo tissues of quinoa seeds, but low amounts in the perisperm. Furthermore, concentrations of essential minerals such as Fe increased due to the high salinity conditions [124]. 

Proteomic and amino acid profiles, phenolic content, and antioxidant activity of protein extracts of seeds from three quinoa varieties, consisting of salares variety ‘R49’ and sea-level varieties ‘VI-1’ and ‘Villarrica’, were analyzed under two salinity levels (100 and 300 mM NaCl) (Aloisi et al., 2016). Results showed a reduction in all amino acids derived from protein hydrolysis in ‘VI-1’ and ‘Villarrica’. However, several amino acids remained unchanged or increased with increasing salinity in ‘R49’. Total polyphenol content increased in the three genotypes with increasing salinity, with the largest increase in the ecoytpe ‘R49’. Similarly, the increases in total flavonoids and total antioxidant activity were more evident in ‘R49’ [116]. In contrast, no change was observed in total polyphenol content in the sprouts of ‘B080’ under 150 mM NaCl conditions; nevertheless, sprout growth was reduced [95].

### 3.5. Gene Expression Under Saline Conditions

Ruiz et al. (2016) described important transcription genes related to the salinity response in quinoa [33]. However, with the recent publication of one robust and two draft quinoa genomes [34,148,166], several new potential genes have been identified that may also play a role in quinoa’s response to salt stress. Table S1 summarizes the genes and candidate genes that have been studied in quinoa under saline conditions.

Exclusion of Na^+^ from the cytoplasm is encoded primarily by two genes. One gene is *Salt Overly Sensitive 1* (SOS1), which encodes a Na^+^/H^+^ antiport located at the plasma membrane of epidermal root cells and functions to extrude Na^+^ out of the cell [167,168,169]. The other gene is tonoplast-localized Na^+^/H^+^ exchanger 1 (NHX1), which sequesters Na^+^ inside the vacuole [170,171]. Maughan et al. (2009) cloned, sequenced, and characterized two homoeologous *SOS1* loci *(cqSOS1A* and *cqSOS1B*) in saline conditions (300 mM NaCl) using the quinoa salares variety ‘Ollague’ [172]. They observed that both genes were up-regulated in the leaves, but not in the roots. Similar results were reported for other quinoa varieties when plants were grown in 300 mM NaCl and 450 mM NaCl [132,173]. However, up-regulation of the gene CqNHX1in shoots and roots was observed in a salt-tolerant variety from Chile when plants were grown in 300 mM NaCl [33,122,132]. In addition, transcription levels of tonoplast intrinsic protein 2 (TIP2) and betaine aldehyde dehydrogenase (BADH) were reported when the two salares varieties ‘Ollague’ and ‘Chipaya’, and one valley variety ‘CICA’, were grown in 450 mM NaCl [173]. Up-regulation of BADH in roots was found in the salares-type genotypes, indicating that betaine plays an important role in decreasing salt stress in roots. Moreover, results revealed that other genes are involved in the mechanisms of the salt stress response [173].

Abscisic acid (ABA), polyamine (PA), and proline biosynthesis genes were studied in two quinoa varieties, ‘R49’ from salares and ‘Villarica’ from sea-level under saline conditions (300 mM NaCl). The expression of 22 genes was common to both varieties. The salt adaptation mechanism was based primarily on ABA-related responses. For example, the gene encoding for the key enzyme in ABA biosynthesis 9-*cis*-epoxycarotenoid dioxygenase (NCED) was the most strongly induced [122]. Likewise, a phylogenetic analysis showed that gene families in ABA signaling were distributed more often in the quinoa genome compared to other *Amaranthaceae* species [166]. Identification of ortholog genes involved in ABA biosynthesis, transport, and perception in quinoa under saline conditions was reported. Hence, quinoa contains neoxanthin synthase (NSY), ABA4, short-chain dehydrogenases/reductases (SDRs) genes, and 11 NCEDs, which are involved in the ABA biosynthetic pathway. The number of these genes in quinoa is nearly two-fold compared to other diploid plants. For example, a higher number of ABA receptor and transportation genes was observed in quinoa, with 22 ABA receptor pyrabactin resistant (PYL) family genes and 81 genes from the ABC transports group (ABCGs) compared to 10 PYL and 34 ABCGs in *Amaranthus hypochondriacus* [148]. 

A transcriptome analysis of bladder cells in quinoa compared a salinity treatment (100 mM NaCl) to non-treated conditions [148]. Results showed a higher expression of genes relative to energy import and ABA biosynthesis in bladder cells compared with the leaf lamina. For instance, anion transporter genes, such as cell anion channels (SLAH), nitrate transporter (NRT), and chloride channel protein (C1C), and cation transporter genes, including NHX1 and K^+^ transporter (HKT1), exhibited a higher expression in bladder cells. After the salt treatment, 180 and 525 differentially expressed genes were identified in leaf lamina and bladder cells, respectively. However, the two tissues shared only 25 genes, indicating that leaf and bladder cells respond differently to salinity [148]. Additionally, genes involved in suberin and cutin biosynthesis are significantly enriched in bladder cells under salinity. On the other hand, photosynthesis and chloroplast protein-encoding genes were down-regulated. The transcript levels of two NCED genes and some of the short-chain SDR genes in bladder cells were six-fold and 1000-fold higher, respectively, than in leaf cells. Furthermore, an elevated expression of ABA transporter and ABA receptor genes was found in bladder cells. Together, the above results suggest that bladder cells might maintain a high level of ABA homeostasis [148]. The ABA biosynthesis pathway shares responsive neoxanthin with an upregulation of NCED genes from both drought and salt stress [148].

RNA-seq analyses with a comparative genomics and topology prediction approach were conducted to identify new transmembrane domain genes in quinoa under saline conditions (300 mM NaCl); 1413 genes were differentially expressed in response to salt [118]. However, 219 genes were chosen after selecting for only genes that encoded proteins with more than one predicted transmembrane domain. These 219 candidate genes were further studied using the sequence information of 14 quinoa varieties (six sea-level, four altiplano, two valley, and two salares), five *C. berlandieri* accessions, and two *C. hircinum* accessions [34], and physiological data under salinity conditions. Using copy number variation (CNV) and the presence of SNPs between the five most salt-tolerant and the five most salt-sensitive accessions, 14 candidate genes were identified, and six SNPs were located in the first exon of the *AUR62043583* gene (Table S1). Thus, the study found 15 new candidate genes that could contribute to the differences in salinity tolerance among quinoa varieties [118]. 

Betalains are tyrosine-derived, red-violet and yellow pigments found exclusively in *Caryopyllales* plants, including quinoa. Betalains are involved in salt stress tolerance due to their antioxidant activity [174]. Mutagenesis using ethyl melthanesulfonate on the quinoa variety ‘CQ127’ revealed that the gene *CqCYP76AD1-1* is involved in the green hypocotyl mutant [175]. This gene was then isolated and shown to be light-dependent in quinoa hypocotyl. These findings suggest that *CqCYP76AD1-1* is involved in betalain biosynthesis during the hypocotyl pigmentation process in quinoa [175]. This gene should be interesting to study under salinity stress because the betalain accumulation could play an important role in protecting quinoa hypocotyl. 

In conclusion, due to its recently sequenced genome and high tolerance to salt stress, quinoa has become an important model crop to further our understanding of how plants respond to salinity. In recent years, using the new molecular tools, several novel genes have been reported. However, validation of these genes is necessary; thus, efforts to transform quinoa have begun to understand the functions of these genes. Furthermore, quinoa’s strong genotype-dependent response to salinity offers breeders the opportunity to work with diverse quinoa genotypes to develop new salt-tolerant varieties with a high grain quality and other valuable traits.

## 4. High Temperature

Excessively high temperature during plant growth is considered one of the most important abiotic stresses, and is being reported with increasing frequency due to the consequences of present-day climate change [176]. Worldwide, extensive agricultural losses have been attributed to heat, often in combination with drought [1,8,177]. Heat stress in plants is defined as an increase in air temperature above the optimum growth temperature for a length of time sufficient to cause damage and, hence, limit growth and development [178]. Heat stress produces different responses across plant species, depending on the temperature duration and the plant developmental stage [179,180].

Effects of heat stress include: (1) morphological changes, such as the inhibition of shoot and root growth and increased stem branching; (2) anatomical changes, such as reduced cell size and increased stomatal and trichome densities; and (3) phenological changes [5,178,181]. In addition to morpho-anatomical changes, physiological effects of heat stress include protein denaturation; increased membrane fluidity; cytoskeleton instability; changes in the respiration, photosynthesis, and activity of carbon metabolism enzymes; osmolyte accumulation; chloroplast and mitochondrial enzyme inactivation; changes in phytohormones, including ABA, salicylic acid, and ethylene; and the induction of secondary metabolites [178,182].

**Figure 3:**
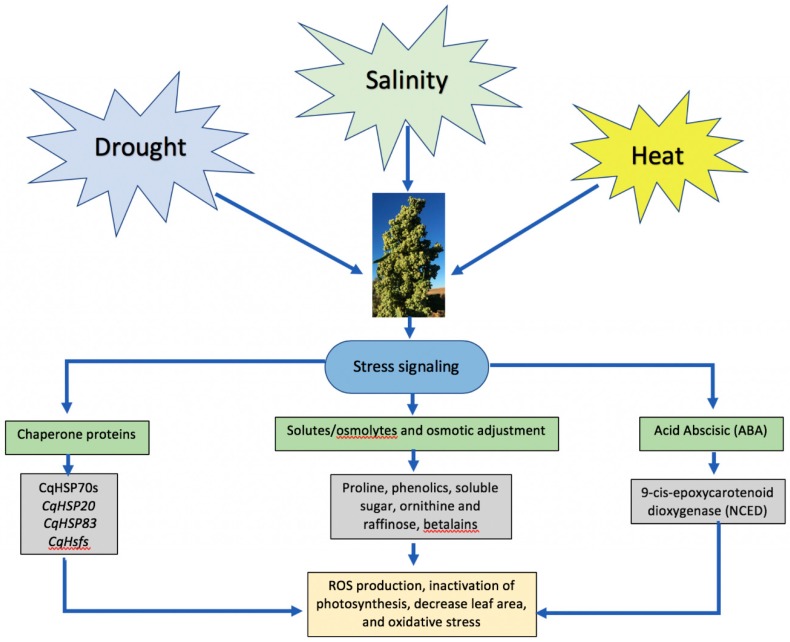
Primary physiological responses to drought, heat, and salinity on quinoa. The expression of NCED genes is upregulated to drought and salinity and chaperone proteins are upregulated to drought and heat stress.

Heat stress induces oxidative-stress-generating ROS, in the same way than in drought or salinity stress, high accumulations of ROS cause serious plant toxicity; however, low concentrations of ROS act as a signaling molecule that activates other plant processes, such as programmed cell death [5,176,178]. Finally, heat-shock proteins (HSPs) play a central role in the heat stress response (HSR) when plants suffer from either an abrupt or gradual increase in temperature [178,183]. Several studies have indicated the importance of HSPs in thermotolerance in many plant species; hence, HSP70 and HSP90 are indispensable to induce thermotolerance [183,184]. Heat stress factors (HSFs) serve as the terminal component of signal transduction of HSP expression. [183,184]. In Figure 3, we show the primary physiological responses to drought, salinity, and heat in quinoa.

Quinoa can tolerate a wide range of temperatures (from −8 °C to 35 °C) and relative humidity conditions (from 40% to 88%), depending on genotype characteristics and phenological stage [185]. Despite the adaptation of quinoa outside the Andes [24,42,43,124,186,187,188,189,190,191,192], a high temperature during flowering and seed set can significantly reduce the yield and is one of the major barriers to the global expansion of quinoa. For example, studies in Italy [45], Morocco [193], Germany [194], Portugal [195], India [27], Egypt [124], Mauritania [42], and the United States [71,196] have reported that high temperatures reduce the quinoa seed yield. Most research has focused on understanding the effect of temperature on quinoa seed germination. However, few studies have focused on understanding the physiological changes in quinoa under high temperature during other phenological stages.

### 4.1. High Temperature Effects on Quinoa Germination

Numerous studies have been carried out in different temperature regimes to describe the effect of temperature on quinoa germination [117,197,198,199,200,201,202,203,204,205]. For instance, studies have found a positive linear relationship between the germination rate and temperature in quinoa [199,202,205]. Findings suggested that the optimal germination temperature is 30 to 35 °C, maximal germination temperature is 50 °C, and base germination temperature is 3 °C [199,202]. In contrast, Bois et al. (2006) reported that the base germination temperatures for 10 different quinoa varieties varied between −1.9 and 0.2 °C [205]. Another study with the variety ‘Titicaca’, and salares varieties ‘Santa Maria’, and ‘Sajama’, used three different models to show that the optimum germination temperature range was 18−36 °C for Sajama and 22−35 °C for the other two varieties [203]. The study also reported a base germination temperature of 1.0 °C and a maximal germination temperature of 54.0 °C for the three varieties evaluated. Quinoa seeds can be stored up to 430 days under controlled environmental conditions, at a constant temperature of 25 °C, before germination completely declines [204]. According to these results, quinoa is highly heat tolerant during its germination stage, and is capable of germinating under a wide range of temperatures, from very low (−1.9 °C) to very hot (>48.0 °C).

### 4.2. High Temperature Effects on Quinoa Growth and Physiological Parameters

The base temperature (T_b_) threshold for quinoa development is variable; for example, in flowering time and leaf appearance, T_b_ is 1 °C, whereas in leaf width, the T_b_ increased to 6 °C [205]. T_b_ can change due to different development rates and the latitude origin of the genotypes [206]; for instance, T_b_ for sea-level variety ‘Baer I’ is 6.4 °C, whereas T_b_ for valley variety ‘Amarilla de Marangani’ is 3.7 °C [207].

Temperatures above 35 °C in the flowering and seed fill stages have been associated with significant reductions in yield. At these temperatures in quinoa fields near Pullman, WA, Peterson and Murphy (2015b) and Walters et al. (2016) observed that inflorescences either lacked seeds or contained empty seeds when the temperature increased above 35 °C [71,196]. Similarly, Bonifacio (1995) observed both the reabsorption of seed endosperm and inhibition of anther dehiscence in quinoa flowers due to high temperature (35 °C) at the flowering stage [208]. However, varietal differences in heat tolerance have been detected in quinoa. For example, sea-level varieties ‘Colorado 407D’, ‘QQ74’, and ‘Kaslaea’ showed greater heat tolerance under field conditions in Pullman compared to other sea-level varieties grown in the same conditions [196].

High night temperatures were evaluated in one commercial sea-level variety, ‘Regalona’, and one quinoa landrace, ‘BO5’, under field conditions in Chile. Results showed that night temperatures between 20–22 °C (~4 °C above the night ambient air temperature) during the flowering stage reduced the seed yield by between 23–31% and negatively affected the biomass and number of seeds. On the other hand, seed protein and harvest index were unaffected [209].

High temperature in quinoa has also been studied in combination with other stresses, such as drought, high salinity, and elevated CO_2_ [54,136,210]. A controlled experiment with ‘Titicaca’ was conducted under cool temperatures of 18/8 °C (day/night temperature) and high temperatures of 25/20 °C, with three different irrigation regimens, consisting of full irrigation, deficit irrigation, and partial root-zone drying [54]. Results showed that drought has a major negative effect on physiological parameters compared to high temperature. In contrast, high temperature increased stomatal conductance, leaf photosynthesis, leaf chlorophyll fluorescence, and chlorophyll content index. On the other hand, anions and cations from the xylem sap increased in response to high temperature, showing that quinoa can adjust osmotically to overcome increased transpirational water. Similar results were observed in stomatal conductance and leaf photosynthesis in altiplano variety ‘Achachino’, grown at 28/20 °C, and sea-level varieties ‘QQ74 (PI 614886)’ and ‘17GR (AMES 13735)’, grown at 40/24 °C [136,211].

**Figure 4:**
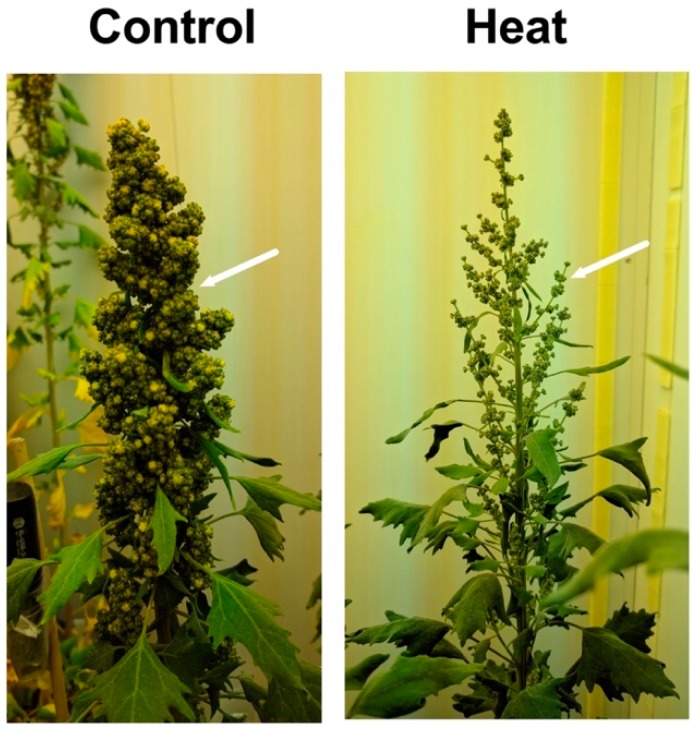
Impact of high temperature on the length of the secondary axis from the quinoa inflorescence. Plant grown under control conditions 22/16 °C (**left**) and high temperature 40/24 °C (**right**).

The altiplano variety ‘Achachino’ was evaluated under high temperature 28/20 °C. Results showed that plant dry mass and yield were unaffected by high temperature; however, more and longer branches were observed in plants due to high temperature [136]. Similar responses were described with quinoa sea-level varieties ‘QQ74’ and ‘17GR’ under 40/24 °C (Figure 4) [211].

Bunce (2017) studied the effect of high temperature and high concentration of CO_2_ in two quinoa sea-level varieties, ‘Red Head’ and ‘Cherry Vanilla’, and one altiplano variety, ‘Salcedo’, during the anthesis stage [210]. Results showed that the harvest index in all varieties either increased or remained the same in response to high temperature (35/29 °C). Seed dry mass decreased in ‘Cherry Vanilla’ when grown at the high temperature and under the ambient CO_2_ concentration (400 μmol·mol^−1^). However, for the other two varieties, when grown under high temperature and either ambient or high CO_2_ concentrations (600 μmol·mol^−1^), seed dry mass was higher than or the same as the control conditions (20/14 °C). Recently, another study grew ‘Cherry Vanilla’ under cool (12/6 °C, 20/14 °C) and moderate (28/22 °C) temperatures. Results showed that this quinoa variety has a large capacity for thermal acclimation to temperature, depending on the maximum carboxylation capacity of Rubisco [212]. However, the effect of high temperature also depends on the variety origin. For example, valley variety ‘Amarilla de Marangani’ produced its heaviest seeds at a day temperature of 20 °C; whereas, quinoa from the sea-level variety, ‘NL-6’, produced its heaviest seeds at a day temperature of 30 °C [186]. Membrane stability of quinoa leaves was measured in six quinoa varieties when grown at 34/32 °C (acclimated) and 22/20 °C (non-acclimated). Results showed that quinoa altiplano variety ‘Illpa’ exhibited less cellular damage after its leaves were exposed to 50 °C for 64 min under acclimated conditions compared to the same exposure under non-acclimated conditions [213]. 

**Figure 5:**
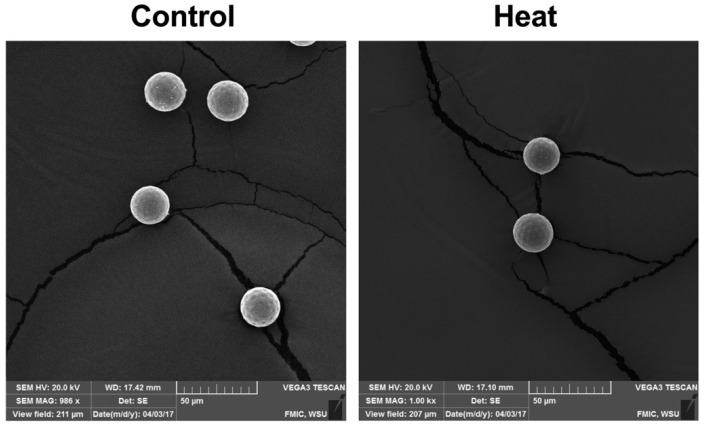
Scanning electron micrographs of quinoa pollen under control conditions 25/16 °C and heat conditions 40/24 °C, magnification 1000×

Quinoa pollen was evaluated for two sea-level varieties, ‘QQ74’ and ‘17GR’, under high temperature (40/24 °C) in growth chamber experiments. Results demonstrated that quinoa pollen viability decreased, but without a concomitant effect on seed set and no morphological changes in the pollen surface (Figure 5). The latter finding was probably due to the high amount of pollen produced by the plants and the high relative humidity (40–65%) recorded in the in the growth chambers [211]. However, field experiments in Pullman, WA-USA, using eight quinoa varieties recorded reduced or total losses in the seed yield, most likely due to the low relative humidity (less than 30%) combined with high temperature (35 °C) and pest pressure.

The heat shock transcription factor (*Hsf*) gene family was studied in quinoa; 23 (*CqHsfs*) genes were identified. The expression profiles from *CqHSFs* genes were explored using RNA-seq data. Four CqHsfs upregulated in the expression profile were then validated in the salar ecotype ‘Real-Blanca’ under high temperature (37 °C), and the results showed that *CqHsfs3* and *CqHsfs9* had a higher expression level after 6 h of heat treatment, while *CqHsfs4* and *CqHsfs10* showed a higher expression level at 12 h [214].

### 4.3. Photoperiod and Temperature Effects on Quinoa

In latitudes greater than 30°, where temperatures exceed 30 °C during the growing season and photoperiods exceed 14 h, quinoa varieties from Andean valleys are low yielding [71,196,215]. In a study with altiplano variety ‘Kanckolla’, the seed diameter decreased as much as 73% when the air temperature rose to 28 °C on long days (16 h) compared to the seeds of quinoa plants grown at 21 °C on short days (10.25 h) [215]. Two models were used to quantify photoperiod and temperature responses in nine short-day varieties from emergence to visible flower buds. Results showed that both models were similar in their goodness of fit. Photoperiod and temperature parameters were not significantly related to latitude of origin; however, a negative association was observed when the attributes evaluated were considered as constants [216]. For the nine varieties evaluated in Bertero et al. (1999b), both temperature and photoperiod controlled the rate of leaf appearance [215]. Temperature sensitivity was the highest for quinoa varieties originating in cold or dry climates, whereas temperature sensitivity was the lowest for varieties from humid and warmer climates [217]. Solar radiation affects phyllochron in quinoa; thus, varieties from Peru, Bolivia, and Southern Chile are more sensitive to the radiation than Ecuadorian varieties. However, Ecuadorian quinoas are highly sensitive to photoperiod and exhibit the longest phyllochron [218]. Saponin content in sea-level varieties ‘Regalona’ and ‘Roja’, and valley variety ‘Tunkahuan’, was evaluated under short days and long days (8 h and 16 h, respectively) and under two temperatures (20 °C and 30 °C) [219]. Results showed that the highest saponin content occurred in plants grown under short days and 30 °C.

In conclusion, quinoa cultivation has expanded worldwide as a crop because of its capacity to thrive in high temperature environments. Although many studies report that plant growth and development are not limited by high air temperatures (~40 °C), reproductive stages can be affected; for example, quinoa pollen viability is reduced at 40 °C. Nevertheless, more studies under both field and semi-control conditions are necessary to evaluate the interactions between high temperature and other abiotic and biotic stressors. Additional studies are also needed that evaluate more genotypes with different planting dates, under tropical and sub-tropical environments, and at different altitudes. Furthermore, wild *Chenopodium* species, such as *C. berlandieri* and *C. hircinum,* are adapted to high temperatures in their native habitats. Thus, another option to develop enhanced heat tolerance in quinoa is interspecific and intergeneric hybridization with these species [220,221,222,223].

## 5. Ultraviolet B (UV-B) radiation

Ultraviolet B (UV-B) radiation represents a small fraction of the solar spectrum (280–315 nm); however, its high energy can be harmful to living organisms [224]. Plants respond differently to UV-B based on their age [225], species origin [226], or circadian rhythms [227]. Studies about UV RESISTANCE LOCUS8 (UVR8) photoreceptor opened the query of whether UV-B should be considered as an abiotic stress or a morphogenetic factor in crop production [228,229,230].

The UV-B effect on quinoa has been mainly studied in South American countries at high altitudes, where UV-B exposure is higher than in other parts of the world [231]. Palenque et al. (1997) reported different responses in morphological and pigment synthesis across quinoa altiplano varieties ‘Chucapaca’, ‘Robura’, and ‘Sayaña’ [232]. They found an increase in leaf flavonoid content and a reduction in plant height and quinoa leaf size in the treatment directly exposed to UV-B; however, ‘Chucapaca’ exhibited the best adaptation to UV-B. Sircelj et al. (2002) revealed the effects of UV-B at metabolic and ultrastructural levels in quinoa seedlings. For instance, the thylakoid organization changed in response to exposure to UV-B [233]. Hilal et al. (2004) found that epidermal lignin deposition in quinoa’s cotyledons was induced by UV-B radiation [234]. Additionally, González et al. (2009) used a semi-control experiment with altiplano varieties ‘Chucapaca’ and ‘Cristalina’ to study the quinoa response to different levels of UV-B [235]. Results demonstrated that sucrose, glucose, and fructose exhibited different distribution patterns in cotyledons and leaves of both varieties, depending on whether exposure was to near-ambient or strongly reduced UV-B. These studies are useful to understand the plasticity of metabolic pathways involved in a plant’s tolerance to solar UV-B radiation.

Another study, under controlled conditions, showed changes in morphological responses, such as plant height, stem diameter, leaf number, and specific leaf area, in different quinoa varieties due to UV-B radiation [236]. The effects of UV-B radiation on photosynthetic (total chlorophyll, chlorophyll *a*, chlorophyll *b*, and carotenoids) and protective (UV-B absorbing compounds) pigments and soluble sugars (glucose, fructose and sucrose) in five quinoa varieties from different geographic origins were studied by Prado et al., (2016) [237]. A common response observed across the five varieties was an increase in the content of UV-B absorbing compounds, which showed a high peak in the absorbance region of 305 nm. The researchers proposed that these compounds act as a “chemical shield” that protect a plant’s photosynthetic apparatus against the excess energy from radiation exposure. 

Recently, Reyes et al. (2018) reported the first study on the effect of UV-B on quinoa photosynthesis [238]. In this study, chlorophyll fluorescence, pigment synthesis, photosynthesis, and ROS accumulation were affected by different levels and duration of UV-B. In summary, quinoa can regulate different mechanisms of response, depending on the UV-B irradiation dosage. Despite the progress in our understanding of the effects of UV-B on plants, additional studies are necessary to: (1) determine the UV-B threshold, where exposure ceases to be a natural morphogenetic factor and becomes a stress factor; and (2) clarify the relationship of UV-B levels with other natural environmental factors.

## 6. Frost, Waterlogging, and Heavy Metals

Other abiotic stressors, such as frost, waterlogging, and heavy metals, have been studied in quinoa [58,185,239,240,241]. Jacobsen et al. (2005) studied different quinoa genotypes under frost temperatures [185]. Results showed that varieties from the Altiplano of Peru tolerated −8 °C for 4 h during the two-leaf stage much better than varieties from Andean valleys, which are more sensitive to frost. For instance, altiplano varieties ‘Witulla’ and ‘Ayara’ experienced a 4.17% plant death rate under −8 °C for 4 h; whereas, valley varieties ‘Quillahuaman’ experienced a 25% death rate under −8 °C for 4 h and 50% under −8 °C for 6 h. Furthermore, the flowering stage is even more sensitive to frost; the study recorded yield reductions of 56% in the variety ‘Quillahuaman’ and 26% in the variety ‘Witulla’ when plants were exposed to −4 °C for 4 h. Another study with ‘Witulla’ and ‘Quillahuaman’ demonstrated that the main quinoa response mechanism to frost is avoidance of ice formation, which is facilitated by the plant’s high soluble sugar content in ‘Witulla’. Thus, proline and soluble sugar contents, such sucrose, could be used as an indicator of frost resistance [240]. Similar results were observed in the altiplano variety Sajama grown under 5/5 °C, where the low temperature induced sucrose-starch partitioning in quinoa cotyledons [242]. Moreover, a low temperature might induce different regulatory mechanisms linked to changes in invertase, sucrose synthase, and sucrose-6-phosphate synthase activity in cotyledons and embryonic axes during Sajama seed development [133].

An experiment in controlled growth chambers with the altiplano variety ‘Sajama’ showed that waterlogging produced several negative effects, including: (1) decreased plant and root dry weights; (2) low total chlorophyll, chlorophyll *a,* and chlorophyll *b* contents; and (3) high amounts of soluble sugars and starch [58]. Under field conditions in Brazil, the variety ‘BRS Piabiru’ exhibited maximum leaf measurement values when plants were grown in a water regimen of 563 mm. However, under 647 mm, reductions in leaf measurements were observed, indicating the sensitivity of quinoa to excess water [241]. Pre-harvest sprouting in quinoa could be a serious problem in places with high precipitation, especially when the rain coincides with the seed-set stage. The quinoa sea-level variety ‘Chadmo, QQ065-PI 614880’ originating from the humid area of Chiloe island in Chile, may be a good choice for these conditions because of its demonstrated higher seed dormancy and greater pre-harvest sprouting tolerance [243,244]. In a comparative study in the high precipitation area of the Olympia Peninsula in Washington State, USA, ‘Chadmo’ showed a higher level of pre-harvest sprouting tolerance [196].

Bhargava et al. (2008b, 2008a) studied different *Chenopodium* spp. under heavy metal soil conditions [3,239]. Results showed that 17 quinoa accessions accumulated high amounts of most heavy metals, such as zinc (Zn), chromium (Cr), nickel (Ni), and cadmium (Cd), in their leaves compared to the other species. For instance, the accessions *C. quinoa* PI 587173, *C. quinoa* PI 478410, C. *quinoa* Ames 22158, and *C. giganteum* CHEN 86/85 accumulated the highest contents of Cd; *C. quinoa* PI 510,536 and Ames 22,156 accumulated the highest contents of Ni, Cr, and Zn. Furthermore, another study in a contaminated urban area ‘brownfield’ in Vancouver, Canada, showed that quinoa from the altiplano variety ‘Quinoa de Quiaca—PI 510532’ is a hyperaccumulator of heavy metals such as Cd, copper (Cu), and lead (Pb). Consequently, quinoa seeds would be inappropriate for human consumption due to high concentrations of trace metals if grown in brownfield areas [245]. A study of quinoa’s physiological response to various concentrations of Cr showed that leaves from sea-level variety ‘Regalona’ tolerated up to 1 mM external chromium(III) chloride (CrCl_3_), activating tocopherol accumulation and enhanced tyrosine aminotransferase content. However, the highest doses of 5 mM Cr(III) produced oxidative stress, generating high hydrogen peroxide and proline contents [246]. Newly discovered heavy metal-related genes involved in the different mechanisms of accumulator plant species and recently published genetic resources could greatly advance novel non-consumptive functions for quinoa. For example, identifying promising functional molecular tools in chenopod species may lead to the effective exploitation of quinoa cultivation as a phytoremediation strategy for environmental contamination cleanup. 

## 7. Conclusions

The physiological, biochemical, and morphological responses of different quinoa varieties to various abiotic stressors, under both field and lab conditions, show that quinoa has a wide plasticity and tolerance to those stressors. This tolerance and plasticity seem to be controlled genetically, and significant advances in breeding have been initiated with the whole genome sequencing of quinoa and the use of new molecular tools. One of the most relevant aspects of quinoa is its high salinity tolerance, unlike other crop species, such as wheat, rice, barley, and maize. Every year, arable land is lost due to salinization, extreme temperatures, and severe drought, which are being reported more often. Hence, farmers have begun to look for halophytic- and abiotic-tolerant species, such as quinoa that can perform under these conditions. Another main feature of quinoa is its high nutritional value, such as essential amino acids and mineral concentrations, which are maintained in spite of abiotic stress conditions. Additionally, quinoa could be considered a multipurpose plant, considering that seeds and leaves can be use as food, biomass can be used as animal feed or a cover crop, and plantings can serve as a phytoremediation tool for environmental cleanup.

Despite the numerous recent studies about abiotic stress on quinoa, much information remains unknown. Future studies should focus on the genetic underpinnings and mechanisms involved in how quinoa’s abiotic stress tolerance influences its chemical composition. This additional information will allow quinoa breeders to generate new varieties that are widely adapted to a variety of environmental conditions, and in turn, facilitate quinoa’s worldwide expansion. Likewise, the recent exploration of intercrosses between quinoa and its wild relatives should provide new genetic combinations with promising opportunities to breed for production in extreme conditions. Taken together, quinoa represents an excellent model to fully explore abiotic stress tolerance mechanisms and new genes to improve plants.

## Supplementary Materials

The following is available online at http://www.mdpi.com/2223-7747/7/4/106/s1, Table S1: Candidate genes involved in the salinity tolerance in quinoa.
