# Supplementary material for: Quinoa Abiotic Stress Responses: A Review
Source: Plants (Basel). 2018 Nov 29;7(4):106. doi: 10.3390/plants7040106 (PMC6313892; doi:10.3390/plants7040106)
Supplement: Supplementary file 1 [file plants-07-00106-s001.pdf]

Table S1. Candidate genes involved in the salinity tolerance in quinoa

Genes	Varieties evaluated and annotations	Salt concentration	Reference
Salt Overly Sensitive 1 (CqSOS1a, CqSOS1b)	Sal variety 'Ollague', up-regulated in leaves but not in roots	300 mM NaCl	[173]
	Sea-level varieties 'PRJ', 'PRP', 'UDEC9', and 'B078'. In shoots strongly up-regulated than in roots	450 mM NaCl	[133]
	Valley variety 'Cica' and the salares varieties 'Ollague', and 'Chipaya'; up-regulated in leaves	450 mM NaCl	[174]
Na^+/H^+ exchanger 1 (CqNHX1)	Sea-level varieties 'PRJ', 'PRP', 'UDEC9', and 'B078'; up-regulated in shoots and roots	450 mM NaCl	[133]
	'Valley variety 'Cica' and the salares varieties 'Ollague', and 'Chipaya'; up-regulation in leaves and shoots	300 mM NaCl	[174]
Betaine aldehyde dehydrogenase (BADH)	'Valley variety 'Cica' and the salares varieties 'Ollague', and 'Chipaya'; up-regulated in leaves	450 mM NaCl	[174]

<table>
<tr>
<td rowspan="3">ABA-related:
9-cis-epoxycarotenoid dioxygenase (NCED)
ABA-binding factors (ABF3)
Pyrabactin resistant (PYR, PYL)
β-glucosidase homologues (BG1)
Polyamine-related
Arginine decarboxylase (ADC1, ADC2)
Spermidine synthase (SPDS1)
S-adenosylmethionine decarboxylase (SAMDC)
Spermine synthase (SPMS)
Diamine oxidase (DAO)
Ion homeostasis-related
CqSOS1a
CqNHX
K^+ transporter (HKT)
Growth:
Cyclin D3 (CycD3)
B-Expansion (βEXP1)
Stress-related genes
Responsive to dessication 22 (RD22)
Pyrroline-5-carboxylate (P5CS)
Transcription factors
Dehydration-responsive element-binding protein 2A (DREB2a)</td>
<td>Salar variety ‘R49’ and sea-level variety ‘Villarica’</td>
<td rowspan="3">300 mM NaCl
0 – 120 hours</td>
<td rowspan="3">[122]</td>
</tr>
<tr>
<td>Salar variety ‘R49’, early up-regulated of ion homeostasis genes and polyamine related genes</td>
</tr>
<tr>
<td>Sea-level variety ‘Villarica’ highly expression on NCED, RD22, and DREB2a</td>
</tr>
<tr>
<td>Pyrabactin resistant (PYR, PYL)
Serine/threonine kinases (SnRK2)</td>
<td>Inbred quinoa accession ‘Kd’. Two phylogenetically similar genes of PYR and two of SnRK2 were observed in quinoa</td>
<td>300 mM NaCl</td>
<td>[167]</td>
</tr>
</table>

Copy number of main genes in salinity conditions: 9-cis-epoxycarotenoid dioxygenase (NCED), 11 genes Neoxanthin synthase (NSY), 7 genes ABA4, 2 genes Short-chain dehydrogenases/reductases (SDRs), 37 genes Pyrabactin resistant (PYL) family, 22 genes Na^+/H^+ exchanger, 11 genes ABC transports group (ABCGs), 81 genes Zeaxanthin epoxidase (ZEP), 2 genes Violaxanthin de-epoxidase (VDE), 2 genes Cell anion channels (SLAH), 6 genes Nitrate transporter (NRT), 12 genes Chloride channel protein (C1C), 10 genes Hemoglobin family (HB), 8 genes H^+ ATPase (AHA), 20 genes Glucose transporter (GLUT), 68 genes	Salar variety 'Quinoa Real' (genome and mRNA sequencing)<br>Quinoa variety 5020 (mRNA sequencing in EBCs)<br>EBC is a photosynthetically inactive tissue and has a strong activity in ion transportation, cell wall and wax synthesis	100 mM NaCl	[151]

Transmembrane domains genes:<br>WAKL8 Wall-associated receptor kinase-like 8 (AUR62006689) (AUR62029668)<br>At1g21890 WAT1-related protein At1g21890 (AUR62039756)<br>At1g67300 Probable plastidic glucose transporter 2 (AUR62021463)<br>CYP75B1 Flavonoid 3’-monooxygenase (AUR62007451)<br>psbD Photosystem II D2 protein (AUR62039871)<br>CER1: Protein ECERIFERUM (AUR62043781) (AUR62043583)<br>AAP6 Amino acid permease 6 (AUR62034957)<br>SULTR1;1 Sulfate transporter 1.1 (AUR62011984)<br>SULTR3;4 Probable sulfate transporter 3.4 (AUR62021522)<br>SULTR3;4 Probable sulfate transporter 3.4 (AUR62016440)<br>CNGC7 Putative cyclic nucleotide-gated ion channel 7 (AUR62004478)<br>DTX14 Protein DETOXIFICATION 14 (AUR62002768)<br>TMK1 Receptor protein kinase 1 (AUR62041961)	14 quinoas (6 sea-level, 4 altiplano, 2 valley, and 2 salares varieties) 5 *C. berlandieri* and 2 *C. hircinum* accessions	300 mM NaCl	[118]
